# Adenylyl Cyclase type 3, a marker of primary cilia, is reduced in primary cell culture and in lumbar spinal cord in situ in G93A SOD1 mice

**DOI:** 10.1186/1471-2202-12-71

**Published:** 2011-07-18

**Authors:** Xiaoxing Ma, Randy Peterson, John Turnbull

**Affiliations:** 1 Department of Medicine, McMaster University, 1200 Main St West, Hamilton, Ontario L8N 3Z5, Canada

## Abstract

**Background:**

The primary cilium is a solitary organelle important in cellular signaling, that projects from the cell surface of most growth-arrested or post-mitotic cells including neurons in the central nervous system. We hypothesized that primary cilial dysfunction might play a role in the pathogenesis of Amyotrophic Lateral Sclerosis (ALS), and as a first step, report on the prevalence of primary cilial markers on cultured motor neurons from the lumbar spinal cord of embryonic wildtype (WT) and transgenic G93A SOD1 mice, and on motor neurons in situ in the lumbar spinal cord.

**Results:**

At 7 days in culture there is no difference in the proportion of G93A SOD1 and WT motor neurons staining for the cilial marker ACIII. However, at 21 days there is a large relative drop in the proportion of ciliated G93A SOD1 motor neurons. In situ, at 40 days there was a slight relative drop in the proportion of ciliated motor neurons in G93A SOD1 mice. At 98 days of age there was no change in motor neuron ciliation in WT mice, but there was motor neuron loss and a large reduction in the proportion of surviving motor neurons bearing a primary cilium in G93A SOD1 mice.

**Conclusions:**

In primary culture and in situ in G93A SOD1 mice there is a large reduction in the proportion of motor neurons bearing a primary cilium.

## Background

Amyotrophic Lateral Sclerosis (ALS) is a neurodegenerative disorder characterized chiefly by progressive and ultimately fatal weakness of voluntary muscle, and is presently defined on clinical grounds. Most cases are without known cause. Some cases are familial (FALS) and of these, a minority are associated with known mutation, in genes encoding SOD1 [1], Alsin [2], Dynactin 1 [3], VAPB [4], angiogenin [5], TDP43 [6,7], or FUS [8,9]. Mutation in genes more commonly associated with other diseases can rarely present as ALS (eg. Kennedy's disease [10], Spastin [11]), as can viral infection (eg. HIV [12] and HTLV [13]). In no case do we fully understand how known mutations lead to ALS, and it is presently unclear how multiple known and unknown triggers can lead to a similar disease phenotype. Several pathological mechanisms may be in common, including impaired axonal transport and reduced trophic support, excitotoxicity, oxidative stress, mitochondrial dysfunction, inflammation, accelerated ageing, errors in RNA processing, and terminally, apoptosis [14]. However, no intervention targeting any of these processes singly has proven successful in substantially mitigating the disease process. It is possible that ALS is complex and will require a multifaceted approach for successful treatment. Alternatively, it is possible that one or several presently unknown causes may lead to a more economical understanding of the disease pathophysiology, and open new avenues for treatment.

The most common model of ALS in present use is the G93A SOD1 mouse, a transgenic model resulting from significant over-expression of a mutant human Cu/Zn superoxide dismutase (SOD1) associated with familial ALS. Overexpression of G93A SOD1 in mice causes a progressive hind limb paralysis, which resembles human ALS in clinical and pathological features [15].

The primary (solitary) cilium is a single microtubule-based organelle that projects from the surface of nearly all post-mitotic or growth-arrested cells and acts as a cellular signaling antenna. Many receptors and ion channels are expressed on the membrane of primary cilia, with expression profiles that differ between organisms and between cell types within an organism. Modified primary cilia on specialized cells transduce the sensory inputs of vision, taste and smell. Intercellular signaling pathways involving the primary cilium include sonic hedgehog (Shh), Wnt, and platelet-derived growth factor (PDGF) among others [16]. These signaling pathways are important in development, and more recently, they have been implicated in cellular homeostasis and resistance to exogenous challenge [17-20]. Previous studies [21] have demonstrated primary cilia on different types of neurons in several species, including granule cells in rat dentate gyrus, major neuron types in rat and guinea pig cerebellum, the hamster paraventricular hypothalamic nucleus, and human neocortex. Immunohistochemical studies using the cilial marker adenylyl cyclase 3 (ACIII) have demonstrated primary cilia on neurons in the mouse hippocampus, cerebral cortex, forebrain, and in the brainstem [22]. The role of primary cilia on spinal motor neurons is less studied.

Since several important signaling pathways converge on the primary cilium, and since some of these pathways are neuroprotective, we questioned whether dysfunction of the primary cilium on spinal motor neurons might be associated with ALS. Accordingly, we used ACIII staining in G93A SOD1 mice to examine the expression of primary cilia on motor neurons in dissociated primary cell culture and in the lumbar spinal cord in situ at two different ages. To date, there has been little work on the expression of primary cilia on motor neurons in the spinal cord even of normal mice, and we are unaware of any studies of primary cilia in hmSOD1 mice. We show here that nearly all motor neurons in the spinal cord of adult wildtype (WT) mice stain avidly for ACIII but motor neurons from embryonic G93A SOD1 mice in primary cell culture, and lumbar motor neurons in situ in G93A SOD1 mice, show greatly reduced expression of ACIII. In the discussion we speculate how absence or dysfunction of the primary cilium might contribute to diseases of the motor neuron.

## Methods

### Mixed primary murine spinal cord cultures

Primary motor neuron cultures were grown from embryonic mice at 14 days gestation, as previously described [23]. Eight to 12 embryos were used per pregnant dam. Briefly, spinal cords were dissected from E14 embryos and were processed individually. A segment of the body was kept for genotyping. Lumbar spinal cord was cut into small pieces and dissociated in 1% trypsin (Sigma) for 15 minutes. After trypsinization an equal volume of trypsin inhibitor (Sigma) was added and the mixture was lightly triturated until a single cell suspension was achieved. The cell suspension was then transferred to Neurobasal medium containing 1% GLUTAMAX (Invitrogen) and centrifuged at 400 g for 5 minutes without brake. The supernatant was discarded and the cell pellet resuspended in complete Neurobasal medium containing 1% GLUTAMAX, 3% horse serum, 1X B-27 supplement (all from Invitrogen), 5 ng/ml ciliary neurotrophic factor (CNTF) and 5 ng/ml brain-derived neurotrophic factor (BDNF) (Leinco). 5 × 10^4 ^cells per chamber were plated on poly-D-lysine (Sigma) coated 8-chamber slides (Labtek) and grown in a 37°C incubator in 5% CO_2 _environment. Half of the culture volume was replaced every third day.

### Immunofluorescent staining of primary cells in culture

At the indicated time points cells were fixed with 4% paraformaldehyde (PFA) at room temperature for 10 minutes, after which cells were washed three times with PBS. Cells were permeabilized with methanol for 10 minutes at -20°C. Fixed permeabilized cells were blocked with 5% goat serum (Invitrogen)/0.3% Triton X-100 (Sigma) in PBS for 1 hour at room temperature. Cells were subsequently hybridized to rabbit anti-ACIII (1:1000, Santa Cruz Biotechnology) and mouse anti SMI 32 (1:1000, Abcam) in 1% goat serum/0.3% Triton X-100 overnight at 4°C. Secondary antibodies used were goat anti-rabbit IgG Alexa Fluor 488 (1:1000, Invitrogen) and goat anti-mouse IgG Alexa Fluor 568 (1:1000, Invitrogen). Secondary antibodies were hybridized in 1% goat serum/0.3% Triton X-100 in PBS at room temperature for 1 hour. Slides were then allowed to air dry in the dark, and then ProLong anti-fade with DAPI (Invitrogen) was added to the slides prior to the addition of the coverslip. Slides were allowed to cure prior to sealing with nail polish.

### Animals and experimental design for in situ analysis of primary cilia

Two age groups, 40 day old and 98 day old transgenic G93A SOD1 mice (B6SJL-TgN[SOD1-G93A]1Gur) were used in the present study. For the 40 day old study, 35 day old animals, WT (littermates, n = 6; 3 males, 3 females) and G93A SOD1 transgenics (n = 5; 3 males, 2 females) were purchased directly from Jackson Laboratory (Bar Harbor, ME), and acclimatized for 5 days before sacrifice. Ninety eight day old animals were taken from a breeding colony established from breeding pairs of male transgenic G93A SOD1 mice bred with female WT B6SJL mice (JAX). Animals, WT (littermates, n = 5; 2 males, 3 females) and G93A SOD1 transgenics (n = 6; 3 males, 3 females) were housed 5 per cage with a 12-h light/dark cycle. All mice were fed standard murine chow and water ad libitum, and food intake was recorded weekly for each cage. Experimental protocols were approved by the McMaster University Animal Research Ethics Board and were carried out in accordance with guidelines of the National Institutes of Health and the Canadian Council on Animal Care.

### Genotyping

DNA was isolated from embryos or from tail snip using a Qiagen blood and tissue preparation kit (Qiagen). 250 ng of total DNA was used to determine the presence or absence of the hmG93A SOD1 transgene using primers and conditions outlined on the JAX website.

### Tissue preparation

Mice were anesthetized with isoflurane inhalation and perfused transcardially with 50 ml of phosphate buffered saline (PBS), followed by 50 ml of 4% PFA. The spinal column was removed and fixed at 4°C overnight, then transferred into a 30% sucrose solution until saturated. Next, lumbar spinal cords were carefully removed using a dissection microscope, frozen, embedded in optimal cutting temperature (OCT) solution, and stored at -80°C until sectioning. A cryostat was used to cut transverse sections, 40 μm, throughout the entire L3 segment of the spinal cord. The L3 segment was identified using the coordinates of Watson et al [24]. Transverse sections were kept at -20°C in a cryoprotectant containing 25% glycerin, 25% ethylene glycol, and 0.05 M phosphate buffer. We examined every sixth section for immunohistochemistry.

### Optimization for double labeling of motor neurons and primary cilia

Commonly used markers to identify motor neurons include anti-Choline acetyltransferase (anti-ChAT) and the 200 kD neurofilament marker SMI 32. In a pilot study, we found that ChAT staining was indistinct in the anterior horns of 98 day old G93A SOD1 mice, and while SMI32 antibody strongly labels neuronal cytoplasmic processes, it is not well suited to counterstaining for a somatic structure like the primary cilium. To circumvent similar problems, previous studies have used a combination of a neuronal cell body stain (eg Nissl) and the size of neurons (>20 μm in diameter) to identify motor neurons [25]. Adopting a similar approach, we used the somatic and nuclear neuronal marker (NeuN) [26] in combination with cell size >20 μm, to identify motor neurons in the ventral horn of the spinal cord. We confirmed that all NeuN positive cells in the ventral horn larger than 20 μm stained for ChAT (Additional file 1).

In a second pilot experiment, we examined the suitability of available primary cilia markers. Some of these did not reliably stain motor neuronal primary cilia, and others, such as acetylated alpha tubulin, stained so diffusely that identification and quantification of primary cilia would have been impossible. Antibodies to both ACIII and melanin-concentrating hormone receptor 1 (MCH1R) stained motor neuronal primary cilia with little background or cytosolic staining, such that reliable counts were possible, and of these ACIII gave the more robust staining. We thus examined primary cell cultures derived from the lumbar spinal cord of embryonic G93A SOD1 and WT mice at 2 time points early and late in culture, as well as the lumbar spinal cord of WT and G93A SOD1 mice before and after the development of signs of paralysis, for the prevalence of ACIII as a marker of ciliated motor neurons. Based on previous observations [27], G93A SOD1 mice at 40 days showed no clinical signs of disease and did not show motor neuron loss in the spinal cord. However, G93A SOD1 mice at 98 days showed clinical signs and obvious motor neuron loss, most particularly in the third lumbar spinal cord segment (L3). This segment corresponds to the spinal innervation of the quadriceps and adductor muscles and is easily identified [28]; as such, this spinal level and these two age groups were chosen to examine the effect of hmSOD1 on cilial expression.

### Immunofluorescent staining

A one-in-six series of sections throughout the entire lumbar L3 segment cut at 40 μm was examined. This resulted in about 5-7 sections examined for the L3 region for each animal. Immunofluorescent double labeling for ACIII and NeuN was done on free floating sections as previously described [29]. Briefly, after rinsing in PBS and blocking with 5% normal goat serum (Vector Laboratories), sections were incubated overnight at 4°C in a cocktail of rabbit polyclonal anti-ACIII (1:200, Santa Cruz Biotechnology, Santa Cruz, USA) and mouse monoclonal anti-NeuN (1:500, Chemicon, Temecula, USA). Next day, sections were rinsed in PBS and incubated for 4 h at 4°C in a cocktail of Alexa Fluor 488 goat anti-rabbit antibody (1:500, Molecular Probes, Carlsbad, USA) and Alexa Fluor 568 goat anti-mouse highly cross-adsorbed antibody (1:500, Molecular Probes, Carlsbad, USA). Sections were then rinsed several times and mounted on slides and coverslipped with ProLong gold antifade reagent with 4,6 diamidino-2-phenylindole (DAPI) (Molecular Probes, Carlsbad, USA).

### Analysis of primary cilia in large and small neurons

Immunofluorescent stained cross-sections at L3 (5-7 sections per animal, 5-6 animals per group) were analyzed with widefield deconvolution microscopy (Leica DMI 6000B, Germany). For each section, images, using a 20X objective lens at multiple consecutive focal planes spaced at 1 μm intervals (Z-stack), were captured with a digital camera (Hamamatsu Orca ER-AG) using Volocity 4 Acquisition Software. The counting areas in the L3 segment derived from Watson et al [24] are shown in Additional file 2. We counted all neurons greater than 20 μm in diameter throughout the Z-stack in the entire lamina IX of the anterior horn on both sides of the cord (as outlined in red on Additional file 2). We counted random fields in lamina VII (as outlined in yellow in Additional file 2), where interneurons reside. No cells > 20 μm were found in this area. Only cells with the nucleus visible were counted. In each section, the number of cells single-labeled with NeuN or double-labeled with NeuN and ACIII was tabulated, and the proportion of double labeled cells to all NeuN labeled cells (ie the proportion of neurons bearing a primary cilium) was calculated.

### Statistical analysis

For the cell culture experiments, the main analysis was the difference in the proportion of motor neurons bearing a primary cilium between cells derived from G93A SOD1 and WT mice, at 7 and at 21 days. This was calculated using the two-tailed z statistic for the difference between proportions, with the usual assumption for this number of samples that the Binomial distribution can be approximated by the Normal distribution. Some observations of the in situ experiment are descriptive in nature; further statistical analysis of the in situ experiment is complex. At a first level of analysis we calculated a Levene's F statistic for the equality of variances (SPSS), and then used a t test for equal or unequal variances (as appropriate), to compare SOD1 and WT animals. This is a commonly accepted approach, but lacks sophistication as the underlying data are proportions, and the actual units of analysis are cells (WT cells or G93A SOD1 cells). Thus, we further analyzed the cell proportion data using binary logistic regression (SPSS), with the presence or absence of a primary cilium as the dependent variable, and neuron size (motor neuron vs interneuron), SOD1 status (WT vs transgenic), age (40 d vs 98 d), and sex (M vs F) as predictive variables, and as well we examined the interaction between predictive variables. Significant differences were defined as p ≤ 0.05, two tailed where applicable.

## Results

### Primary neuronal cultures

**Figure 1:**
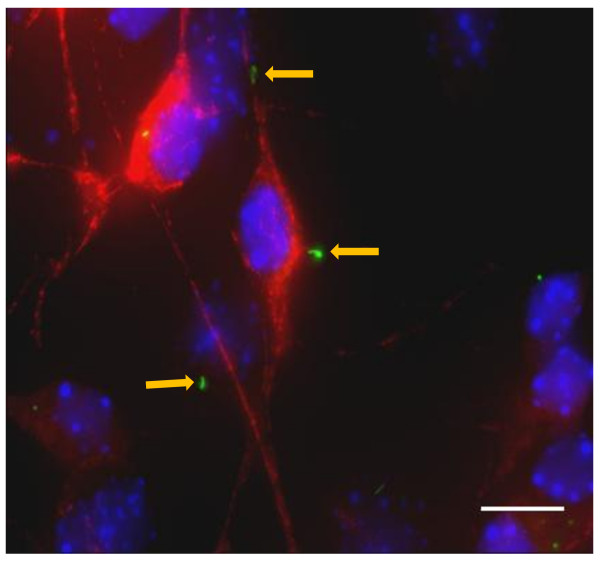
**Cells from embryonic mouse lumbar spinal cord in primary mixed culture**. Motor neurons are stained with SMI32 (red), primary cilia are stained with ACIII (green), and the nuclear stain DAPI is blue. In the center of the photomicrograph, a ciliated motor neuron is seen. Above it and below it, two cilia are seen, not associated with motor neurons. Scale Bar = 10 μM. Yellow arrows indicate ACIII labeled primary cilia

**Figure 2:**
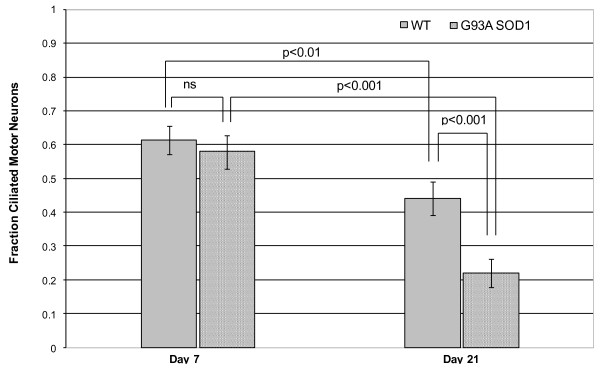
**The fraction of ciliated motor neurons in WT and G93A SOD1 primary culture, at 7 and 21 days**. The fraction of ciliated motor neurons in WT and G93A SOD1 primary culture, at 7 days and at 21 days in culture. There is a decline in the proportion of ciliated motor neurons with increasing age in culture, especially in G93A SOD1 transgenic cells. ns: p < 0.05.

Primary cilia on motor neurons in culture could be robustly identified by co-staining with SMI32 and ACIII (figure 1). (Throughout this paper, we have used the term co-localization to imply the co-localization of cilial and neuronal stains to the same cell). After 7 days in mixed primary culture, the percentage of cells staining positive for SMI32 (motor neurons) did not differ between WT cells (13.9 ± 4.62%) and G93A SOD1 cells (12.8 ± 3.66%). Sixty one of 100 WT cells staining positive for SMI32 expressed a primary cilium, compared with 57 of 100 G93A SOD1 cells (p = 0.56). At 21 days, there was no difference in the percentage of motor neurons in cultured WT (12.3 ± 2.35%) and G93A SOD1 cells (9.5 ± 4.15%). There was a drop in ciliated motor neurons, especially in G93A SOD1 cells (44 of 100 WT cells positive for SMI32 expressed a primary cilium, vs 22 of 100 G93A SOD1 cells, p = 0.001). This is illustrated in figure 2. Interestingly, this drop was seen more in motor neurons than other cell types in these mixed cultures (not shown).

### In situ distribution of ACIII positive primary cilia

Since no previous reports have discussed ciliated cells in the G93A SOD1 mouse lumbar cord, we first examined the general distribution of ACIII labeling on all cells in the L3 spinal cord, for both WT and G93A SOD1 mice at 40 and 98 days of age. At low magnification, primary cilia were detected on cells throughout L3, including in spinal laminae I through X (Additional file 3a, c). Ciliated cells were particularly abundant in laminae I-VI of dorsal horns and lamina VII of ventral horns. At higher magnification, primary cilia typically displayed a rod-like shape with bright and homogenous staining for ACIII (Additional file 3b, d.). Although the length of primary cilia was not formally measured, it was apparent that the length of the primary cilium varied across regions of the spinal cord, with longer primary cilia in spinal laminae V, VI, VII, VIII and shorter cilia in spinal laminae I, II, III, IV, and IX. Thus, there is regional heterogeneity of primary cilia with respect to length, as previously reported by Fuchs [21] in an ultrastructural study. The general appearance and general distribution of ACIII did not differ between WT mice and G93A mice, at either age.

### Primary cilia project from neurons

**Figure 3:**
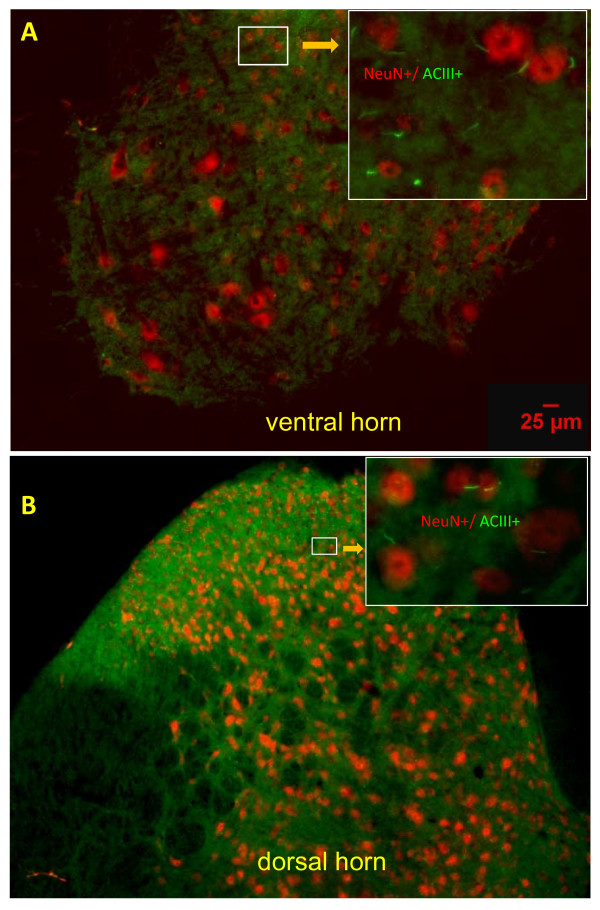
**ACIII labeled primary cilia project from NeuN labeled neurons in the spinal cord**. Representative microscopic images show overall co-localization of ACIII (green) and NeuN (red) in the ventral horn (A) and dorsal horn (B) of L3 spinal cord in 98d-old WT mice. At higher power, inserts in A and B show numerous ACIII labeled rod-like primary cilia (green) projecting from NeuN labeled neurons (red).

We studied the co-localization of ACIII staining with NeuN staining, in the spinal cords of WT and G93A SOD1 mice at both age groups. Most NeuN positive cells co-stained with ACIII in the spinal cord, for both WT and G93A SOD1 mice, at both ages. Figures 3 showed representative low magnification microscopic channel merged images of double stained (ACIII labeled primary cilia, dot-like structures in green, and NeuN positive neurons labeled in red), in the ventral and dorsal horn of L3 of WT 98 day old mice. At higher magnification, most of ACIII positive primary cilia, rod-like structures in green, were shown to co-localize with red NeuN staining, for all mice (figure inserts in 3a and 3b). Thus, most neurons throughout the spinal cord possess a primary cilium, and most primary cilia are associated with neurons.

**Figure 4:**
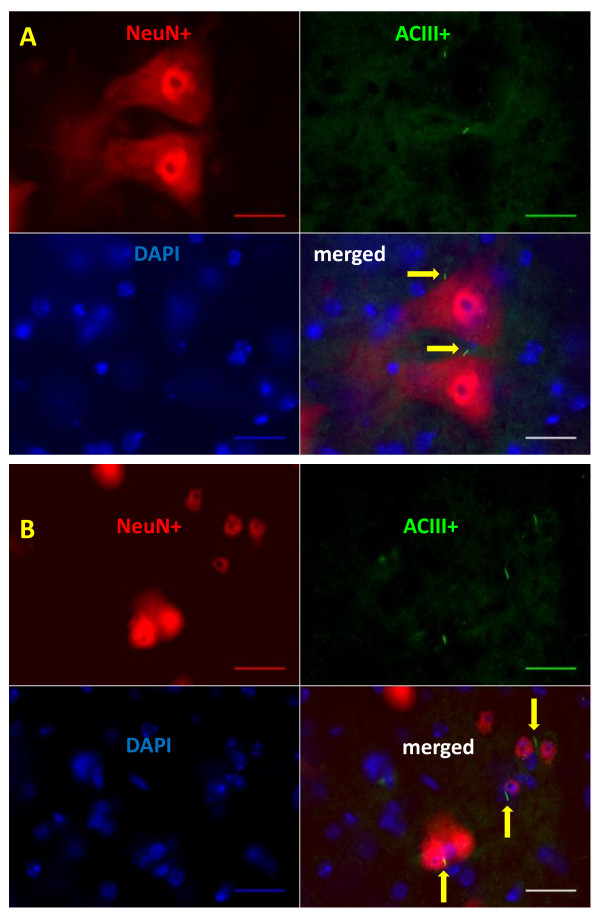
**Ciliated motor neurons and ciliated small neurons in 98d WT mice**. A photomicrograph of ACIII labeled primary cilium (green), NeuN labeled neurons (red), and two motor neurons double labeled with ACIII and NeuN (merged channel) are shown in the ventral horn of a WT-98d mouse (A). In the lower panel, ACIII labeled primary cilia were co-localized with NeuN small neurons in lamina VII of WT-98d mice. Scale Bar = 25 μM. Yellow arrows indicate ACIII labeled primary cilia.

In lamina IX, most NeuN positive cells are large (> 20 μm). That is, most neurons are motor neurons. Figure 4a shows representative images of large motor neurons co-localized with ACIII labeled primary cilia (yellow arrow), in WT 40 day and WT 98 day mice. In laminae V, VI, VIII, VIII, most neurons are small (< 20 μm), and most of these small NeuN labeled neurons co-labeled with ACIII (Figure 4b), suggesting that interneurons possess primary cilia. Occasionally small NeuN positive cells did not co-label with ACIII. Also, some ACIII positive cells did not co-stain with NeuN, suggesting that ACIII labeled primary cilia may project from glial cells.

### Quantification of primary cilia in Lamina VII

At 40 days there is no difference in the average number of L3 neurons (NeuN positive cells) in lamina VII between WT and G93A SOD1 mice (158.2 ± 29.4 vs 162.2 ± 14.3; p = 0.79). There is no difference in the proportion of ciliated neurons between WT and SOD1 mice (0.93 ± 0.031 WT vs 0.91 ± 0.040 SOD1; p = 0.65 using t test for equal variances (F = 0.151; p = 0.71)). The proportion of ciliated small neurons for each animal at 40 days is shown in Additional file 4.

At 98 days the number of small neurons was slightly less than at 40 days, and in addition there was a slight non-significant reduction in neuron numbers in SOD1 as compared to WT mice (120.4 ± 24.1 vs 86.5 ± 21.5; p = 0.34). There was a modest relative decline in the proportion of ACIII and NeuN double labeled cells in G93A SOD1 mice that did not reach significance (0.96 ± 0.012 WT vs 0.81 ± 0.073 SOD1; p = 0.09 using t test for unequal variances (F = 11.8; p = 0.007)). The proportion of ciliated small neurons at 98 days is shown for each animal in Additional file 5.

### Quantification of primary cilia on motor neurons in lamina IX

**Figure 5:**
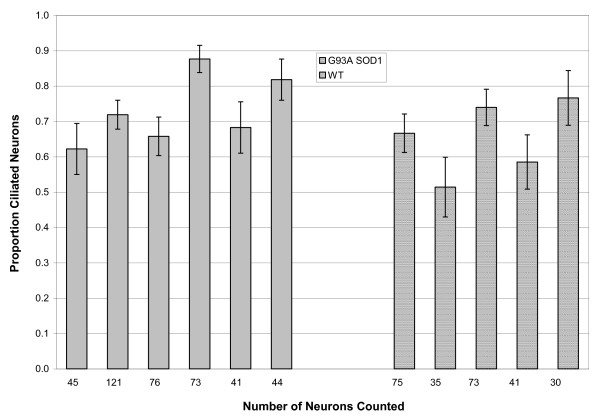
**Ciliated motor neurons at 40d in WT and G93A SOD1 mice**. The proportion ± SD. of ciliated motor neurons at L3 is shown for WT (n = 6; 3 males, 3 females) and G93A SOD1 (n = 5; 3 males, 2 females) mice, at 40 days of age. The number of motor neurons comprising the sample is shown under the bar for each animal.

At 40 days, there was no difference in average L3 anterior horn motor neuron number between WT and G93A SOD1 mice (66.6 ± 12.5 WT vs 50.8 ± 8.8 SOD1; p = 0.36). However, there was a non-significant reduction in the proportion of motor neurons bearing a primary cilium in the G93A SOD1 group (0.73 ± 0.041 WT vs 0.65 ± 0.048 SOD1; p = 0.267 using t test for equal variance (F = 0.026; p = 0.876)). The proportion of ciliated motor neurons is shown for each animal in figure 5.

**Figure 6:**
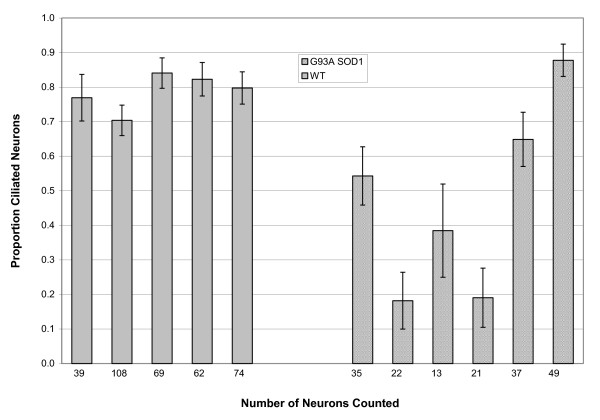
**Ciliated motor neurons at 98d in WT and G93A SOD1 mice**. The proportion ± SD. of ciliated motor neurons at L3 is shown for WT (n = 5; 2 males, 3 females) and G93A SOD1 (n = 6; 3 males, 3 females) mice, at 98 days of age. The number of small neurons comprising the sample is shown under the bar for each animal.

**Figure 7:**
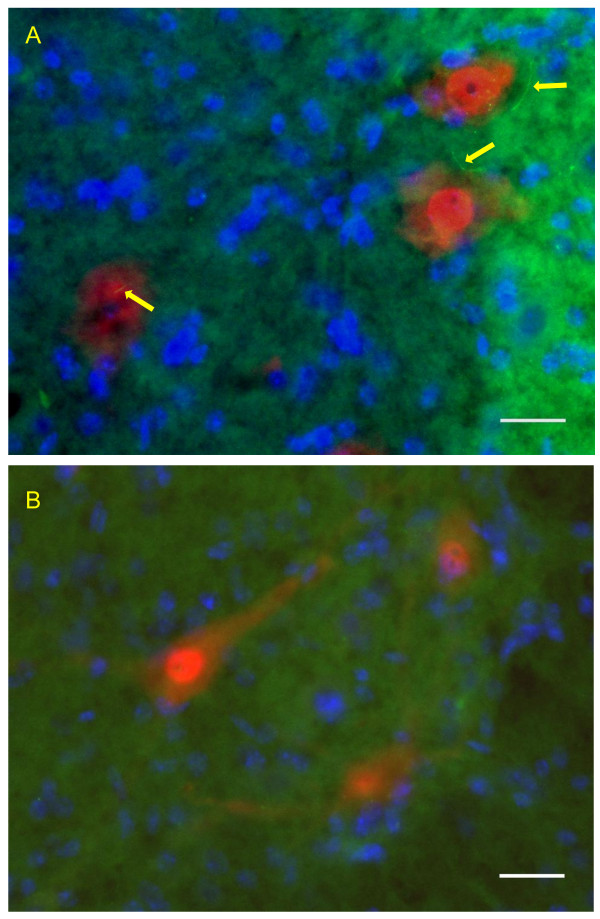
**Reduced ACIII labeled primary cilia in the ventral horns of 98d G93A mice**. A representative photomicrographs showed that in a WT mouse where all three motor neurons bear primary cilia (figure 7a), whereas none among three motor neurons had a primary cilium in a G93A SOD1 mouse (figure 7b). Scale Bar = 25 μM. Yellow arrows indicate ACIII labeled primary cilia.

At 98 days, there was a significant reduction in average L3 motor neuron numbers between WT and SOD1 animals (70.4 ± 11.1 vs 29.5 ± 5.4; p = 0.006). In addition, there was a major reduction in the proportion of remaining motor neurons bearing a primary cilium (0.79 ± 0.02 WT vs 0.47 ± 0.11 SOD1; p = 0.03 using t test for unequal proportions (F = 8.837; p = 0.016)). The proportion of ciliated motor neurons for each animal at is shown graphically in figure 6. This is also illustrated in representative photomicrographs, in a WT mouse where all three motor neurons bear primary cilia (figure 7a), whereas none among three motor neurons had a primary cilium in a G93A SOD1 mouse (figure 7b). There is considerably more variability in the cilial proportions in G93A SOD1 mice, as evidenced by the F statistics above, with marked variability even within different regions within the same animal and larger standard deviations in the proportions.

A logistic regression of this dataset, with presence or absence of a cilium as the dependent variable, and age, transgenic status, neuron size (ie >20 μm), and sex as predictive variables, shows that presence of a mutant transgene is a highly significant predictor of reduced ciliation (p < 0.001), as is large neuron size (p < 0.001). There is a strong interaction between transgene status and age (p < 0.001; reflecting the drop in ciliation in aged transgenics but not in aged WT mice). Otherwise, age adds no independent predictive value (p = 0.248). Female sex is minimally and non-significantly predictive (p = 0.165) of greater ciliation.

## Discussion

It is common practice to demonstrate primary cilia using immunohistochemical staining for ACIII [22,30], and on this basis we conclude that over-expression of a disease-causing SOD1 mutation significantly reduces the expression of primary cilia on motor neurons late in primary culture and in the lumbar spinal cord of adult animals in situ. However, it might be more conservative to conclude that there is a reduction in ACIII cilial staining, as it is theoretically possible that the ACIII staining has disappeared from an otherwise intact primary cilium. There could be important consequences in either event, and moreover, since our study focused on immunohistochemical alterations only, the potential importance of these findings would be broadened if there were additional deficits in cilial function in motor neurons still retaining ACIII staining.

Some technical issues bear discussion. Because of restrictions imposed by antibody suitability, species, and the availability of secondary stains, we chose to identify motor neurons indirectly. Usual stains for motor neurons, eg SMI32 or ChAT, result in a stain that is diffused and poorly suited to counterstaining for cilia. In consequence, we used an indirect method for identifying ciliated motor neurons. We first verified that nearly all large neurons (greater than 20 μm) in the anterior horn co-express ChAT and the neuronal stain NeuN, and then used NeuN, which is better suited to the identification of cilia, and size greater than 20 μm, as a surrogate for direct identification of motor neurons. The indirect identification of motor neurons could introduce error if some motor neurons in the hmSOD1 mice were pyknotic (and thereby not identified as motor neurons by size). However, there were very few NeuN positive cells less than 20 μm in lamina IX in either WT or G93A SOD1 mice, and thus pyknotic motor neurons would have to have lost their NeuN staining (which has been reported in neurons in an experimental stroke model [26]).

We used 40 μm sections and only counted cells where the nucleus could be identified in totality by imaging at multiple planes. It is possible that a primary cilium was present on some cells but out of section, and the cell thus misidentified as lacking a primary cilium. This error would not be large, and indeed, if there were somatic shrinkage with disease, it would bias against finding a difference in G93A SOD1 mice.

Even in diseased animals, some neurons appeared healthy and had normal appearing primary cilia, and thus there was considerable variability in cilial staining. This variability raises the possibility that the disease process is patchy with respect to cilial pathology, that there are differences in susceptibility between types of motor neurons, or that there is a differential expression of hmSOD1 between individual motor neurons in this model. (There is some evidence that the transgene copy number may not be fully stable in G93A SOD1 mice [31] but nothing is known of cellular mosaicism.)

In spite of these caveats, our cell culture results and our animal studies are mutually consistent, and consistent with the hypothesis that there is a reduction in the proportion of ciliated motor neurons in G93A SOD1 mice. Neurotoxic SOD1 mutations could affect primary cilial structure and function in several ways. There might be intra-ciliary protein accretion and/or altered intraflagellar transport, and in this light, mSOD1-induced changes in the normal anatomical barriers to the exchange of proteins between cytosolic and ciliary compartments might be important (ie the ciliary necklace and transitional fibers [32]).

A major unanswered question that will await further experimentation is whether the reduction in ciliated motor neurons simply reflects the underlying diseased state of these cells, or whether the loss may be more directly and causally related to the ALS disease process. There are several reasons to believe that a loss of primary cilia could be detrimental. Most directly related to this work, the loss of ACIII would be expected to disrupt cAMP second messenger signaling, in this case from an unknown G protein coupled receptor and unknown ligand [33].

Moreover, much, indeed perhaps all, Shh signaling occurs through the primary cilium, and disruption of the primary cilium alters the balance between canonical and non-canonical Wnt pathways [16]. Both Shh and Wnt have been shown to exert neuroprotective function [17-20,34,35]. Similarly, other signaling pathways involve the primary cilium and may be neuroprotective, including MCH1, SSTR3, and PDGF [36-38]. Loss of cilial signaling (or reduced cilial signaling due to reduced cilial stability and increased cilial turnover) could render motor neurons more susceptible to neurotoxic challenge.

Last, the primary cilium derives from the maternal kinetophore, is expressed only on growth-arrested cells [16] (possibly for this reason), and perhaps not surprisingly many cilial signaling pathways have been implicated in cell cycle control. Specifically, Shh, Wnt, PDGF, and SSTR3 have all been implicated in cell cycle control [39-43]. Disruption of primary cilial function might alter the response of post-mitotic motor neurons to disease-induced cell cycle re-entry signals [44,45], with possible adverse consequences [46].

## Conclusions

Primary cilia are abundant and widely distributed in motor neurons in primary culture from normal embryonic mice and in the lumbar spinal cord of adult mice. In G93A SOD1 mice, the proportion of ciliated motor neurons drops markedly in primary culture, and in the lumbar spinal cord in situ. Future experiments will determine whether alterations in primary cilia contribute to the pathogenesis of ALS, and by extension, of other neurodegenerative diseases.

## Authors' contributions

XM performed all the in situ work, including all the in situ stains, counts, and photomicrographs, and analyzed this aspect of the paper. She contributed to writing the paper. RP cultured the primary neurons, performed all the cilial counts in culture, took the photomicrographs of cultured cells, and performed the analysis of these data. He contributed to writing the paper. JT conceived the experiments and largely wrote the paper. All authors read and approved the final manuscript.

## Supplementary Material

Additional file 1 **Co-localization of ChAT and NeuN staining in motor neurons**. A representative image showing colocalization of ChAT (green) and NeuN (red) staining in large (>20 μm) cells in the ventral horn of L3 of 98d WT mice. Scale bar 25 μm. Click here for file

Additional file 2 **Sampling areas in the L3 cord**. The L3 cord was used to draw the sampling areas (shown on one side for illustration purposes but counted on both sides), using the mouse atlas of Watson et al. Motor neurons were located in lamina IX, outlined in red, and interneurons in lamina VII (yellow). Click here for file

Additional file 3 **Distribution of ACIII staining for primary cilia in L3 cord**. Representative images show that ACIII labeled primary cilia (dot or rod-shaped structures in green) are widely distributed in the ventral horn (A) and in the dorsal horn (C) of wild type 98d mice. Red arrows indicate ACIII labeled primary cilia. Click here for file

Additional file 4 **Ciliated small neurons at 40d in WT and G93A SOD1 mice**. The proportion ± SD. of ciliated small neurons at L3 is shown for WT (n = 6; 3 males, 3 females) and G93A SOD1 (n = 5; 3 males, 2 females) mice, at 40 days of age. The number of small neurons comprising the sample is shown under the bar for each animal. Click here for file

Additional file 5 **Ciliated small neurons at 98d in WT and G93A SOD1 mice**. The proportion ± SD. of ciliated small neurons at L3 is shown for WT (n = 5; 2 males, 3 females) and G93A SOD1 (n = 6; 3 males, 3 females) mice, at 98 days of age. The number of small neurons comprising the sample is shown under the bar for each animal. Click here for file
