# Supplementary figures and images for: Adenylyl Cyclase type 3, a marker of primary cilia, is reduced in primary cell culture and in lumbar spinal cord in situ in G93A SOD1 mice
Source: BMC Neurosci. 2011 Jul 18;12:71. doi: 10.1186/1471-2202-12-71 (PMC3199874; doi:10.1186/1471-2202-12-71)

## Slide 1
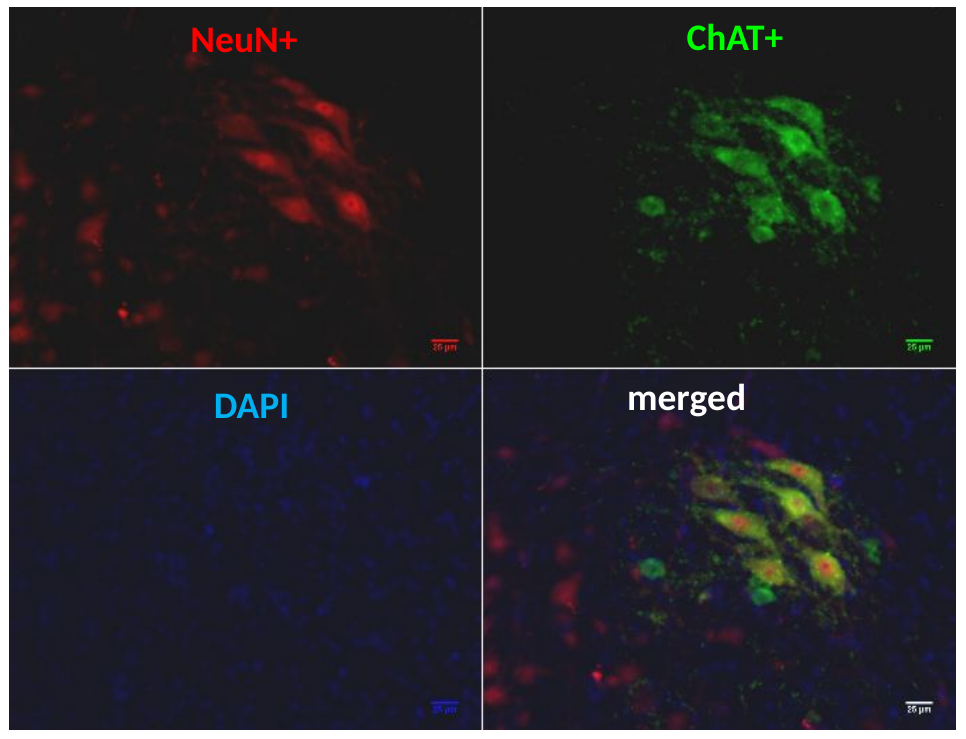

ChAT+
NeuN+
merged
DAPI

Supplement: Additional file 1 — Co-localization of ChAT and NeuN staining in motor neurons. A representative image showing colocalization of ChAT (green) and NeuN (red) staining in large (>20 μm) cells in the ventral horn of L3 of 98d WT mice. Scale bar 25 μm. [file 1471-2202-12-71-S1.PPT]

## Slide 1
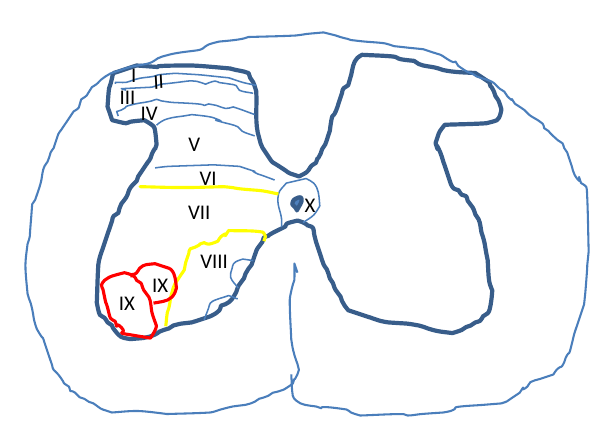

I
II
III
IV
V
VI
X
VII
VIII
IX
IX

Supplement: Additional file 2 — Sampling areas in the L3 cord. The L3 cord was used to draw the sampling areas (shown on one side for illustration purposes but counted on both sides), using the mouse atlas of Watson et al. Motor neurons were located in lamina IX, outlined in red, and interneurons in lamina VII (yellow). [file 1471-2202-12-71-S2.PPT]

## Slide 1
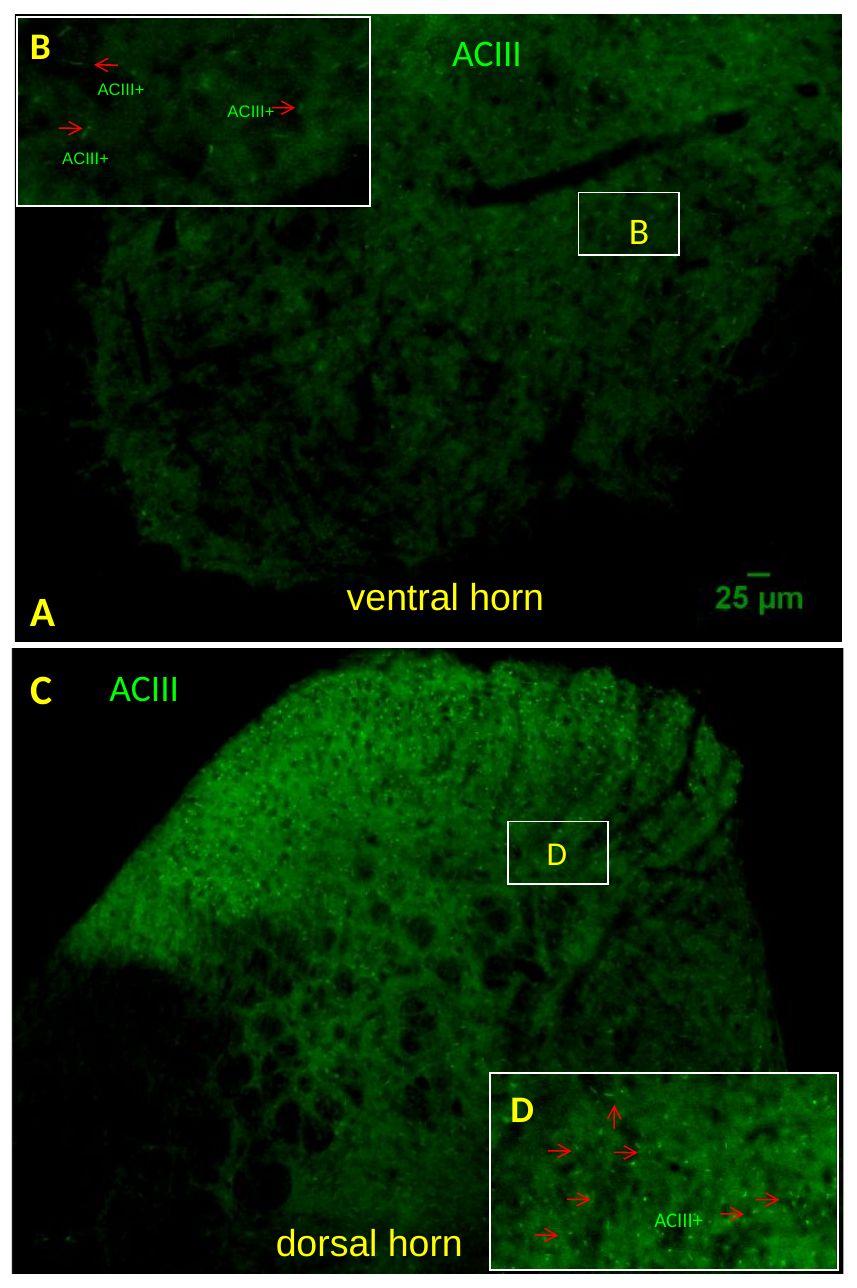

B
ACIII
ACIII+
ACIII+
ACIII+
B
ventral horn
A
C
ACIII
D
D
ACIII+
dorsal horn

Supplement: Additional file 3 — Distribution of ACIII staining for primary cilia in L3 cord. Representative images show that ACIII labeled primary cilia (dot or rod-shaped structures in green) are widely distributed in the ventral horn (A) and in the dorsal horn (C) of wild type 98d mice. Red arrows indicate ACIII labeled primary cilia. [file 1471-2202-12-71-S3.PPT]

## Slide 1
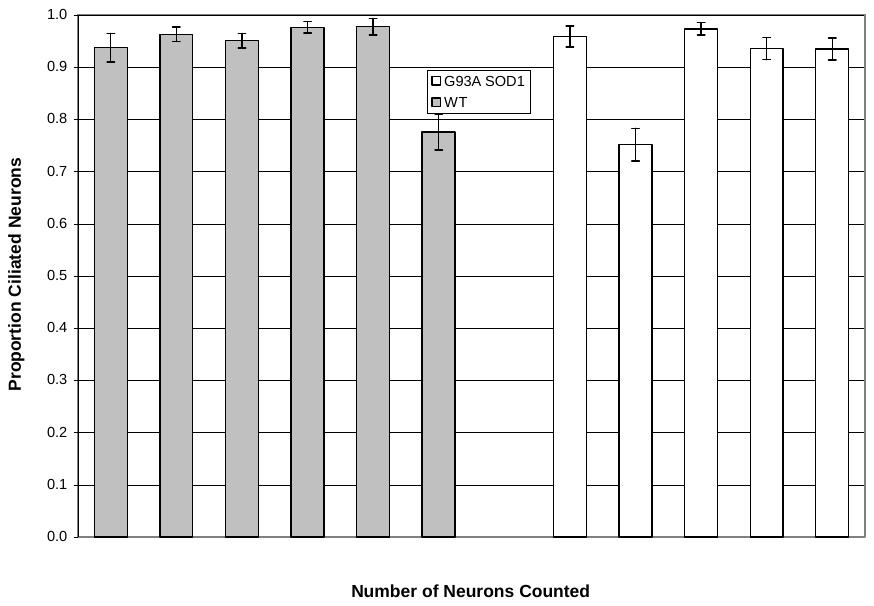

Supplement: Additional file 4 — Ciliated small neurons at 40d in WT and G93A SOD1 mice. The proportion ± SD. of ciliated small neurons at L3 is shown for WT (n = 6; 3 males, 3 females) and G93A SOD1 (n = 5; 3 males, 2 females) mice, at 40 days of age. The number of small neurons comprising the sample is shown under the bar for each animal. [file 1471-2202-12-71-S4.PPT]

## Slide 1
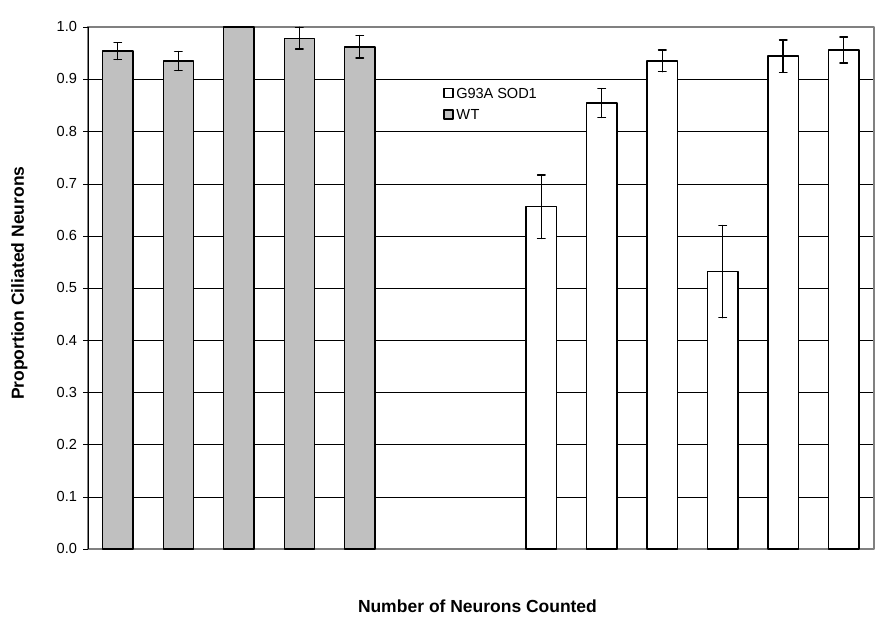

Supplement: Additional file 5 — Ciliated small neurons at 98d in WT and G93A SOD1 mice. The proportion ± SD. of ciliated small neurons at L3 is shown for WT (n = 5; 2 males, 3 females) and G93A SOD1 (n = 6; 3 males, 3 females) mice, at 98 days of age. The number of small neurons comprising the sample is shown under the bar for each animal. [file 1471-2202-12-71-S5.PPT]
